# Measuring benefits and patients' satisfaction when glasses are not needed after cataract and presbyopia surgery: scoring and psychometric validation of the Freedom from Glasses Value Scale (FGVS^©^)

**DOI:** 10.1186/1471-2415-10-15

**Published:** 2010-05-24

**Authors:** Gilles Berdeaux, Juliette Meunier, Benoit Arnould, Muriel Viala-Danten

**Affiliations:** 1 Health Economics Eurmea, Alcon Laboratories, 4, rue Henri Sainte-Claire Deville, 92563 Rueil-Malmaison Cedex, France; 2 Statistics and Psychometrics, Mapi Values, 27, rue de la Villette, 69003 Lyon, France

## Abstract

**Background:**

The purpose of this study was to reduce the number of items, create a scoring method and assess the psychometric properties of the Freedom from Glasses Value Scale (FGVS), which measures benefits of freedom from glasses perceived by cataract and presbyopic patients after multifocal intraocular lens (IOL) surgery.

**Methods:**

The 21-item FGVS, developed simultaneously in French and Spanish, was administered by phone during an observational study to 152 French and 152 Spanish patients who had undergone cataract or presbyopia surgery at least 1 year before the study. Reduction of items and creation of the scoring method employed statistical methods (principal component analysis, multitrait analysis) and content analysis. Psychometric properties (validation of the structure, internal consistency reliability, and known-group validity) of the resulting version were assessed in the pooled population and per country.

**Results:**

One item was deleted and 3 were kept but not aggregated in a dimension. The other 17 items were grouped into 2 dimensions ('global evaluation', 9 items; 'advantages', 8 items) and divided into 5 sub-dimensions, with higher scores indicating higher benefit of surgery. The structure was validated (good item convergent and discriminant validity). Internal consistency reliability was good for all dimensions and sub-dimensions (Cronbach's alphas above 0.70). The FGVS was able to discriminate between patients wearing glasses or not after surgery (higher scores for patients not wearing glasses). FGVS scores were significantly higher in Spain than France; however, the measure had similar psychometric performances in both countries.

**Conclusions:**

The FGVS is a valid and reliable instrument measuring benefits of freedom from glasses perceived by cataract and presbyopic patients after multifocal IOL surgery.

## Background

Cataracts and presbyopia are both conditions that cause visual impairment. Cataracts, responsible for almost half the cases of blindness worldwide [1], are cloudy areas of accumulated protein that form on the lens of the eye, causing blurred and reduced vision, faded color perception, glare, light sensitivity and impaired night vision. Presbyopia is the loss of the natural lens' flexibility, which makes it difficult to focus on close objects. Age is the major cause of development for both cataracts and presbyopia. Many aspects of patients' daily lives are affected by these two conditions, and there is increasing interest in evaluating their impacts on patients' quality of life [2-6].

Surgery, consisting of the replacement of the natural lens by an intraocular lens (IOL), is the only effective treatment for cataracts [7]. More recently, presbyopia-correcting lenses have been used to treat presbyopia in patients undergoing cataract surgery [8,9]. The benefits of cataract surgery for patients' visual functioning, satisfaction and quality of life are well-documented in the literature [4,10-16]. Monofocal IOL implantation allows either distance or near vision to be corrected, so patients with both problems still need to wear glasses to correct the one or the other visual impairment. Multifocal IOLs were then developed to restore both distance and near vision and, consequently, to free patients from glasses.

Several multifocal intraocular implants have been specifically designed as an option for cataract surgery lens replacement, as well as for patients with presbyopia. Their safety and efficacy, as well as benefit for visual functioning and patient satisfaction, are well-reported in the literature [10,14,16-25]. However, the value for patients of no longer requiring glasses has not been specifically documented. Typical measures used to assess the benefit of IOLs include clinical parameters, a functional test of visual acuity and some functional Patient-Reported Outcome (PRO) scales (e.g. TyPE questionnaire [26], Visual Function Index (VF-14) [27]). Some other PRO scales were specifically designed to measure the impact of assistive devices or correction - either with glasses or contact lenses - on patients' quality of life (e.g. Quality of Life Impact of Refractive Correction questionnaire (QIRC) [28], Contact Lens Impact on Quality of Life (CLIQ) [29], Psychosocial Impact of Assistive Devices (PIADs) [30]). To our knowledge, no existing PRO scales assess the perceived patient benefit of freedom from glasses. A specific questionnaire, the Freedom from Glasses Value Scale (FGVS^©^), was developed [31] for this purpose and administered to French and Spanish patients during an observational study.

The objectives of this paper are to present the item reduction process and creation of the scoring method for the FGVS, and then to assess the psychometric properties of the resulting version of the measure.

## Methods

### The FGVS

The FGVS was developed simultaneously in French and Spanish following patient interviews and the development of a conceptual framework comprising 9 global concepts identified from analysis of the exploratory patient interviews [31]. Based on these concepts, 21 items were generated: global vision (1 item), impact of eye surgery on patients' lives (1 item), practical constraints related to wearing glasses (1 item), improvement of practical issues without glasses (8 items), improvement of psychological constraints without glasses (5 items), physical appearance/aesthetic aspect (self image) (1 item), physical appearance/aesthetic aspect (in the eyes of others) (1 item), eyesight problems left behind (1 item) and recommendation of surgery to others (2 items). All items had 5-point Likert response scales, ranging from "much better" to "much worse", "very positive" to "very negative", "no, not at all" to "yes, absolutely", "totally agree" to "totally disagree" or "definitely better without glasses" to "definitely better with glasses".

### Patients and study design

An observational, cross-sectional, non-comparative, multicentre study was conducted in France and Spain between June 2007 and January 2008 involving patients who had a ReSTOR^® ^lens implanted in both eyes, with the last eye operated at least 1 year before the inclusion date. Centres were selected from each country in which surgeons were experienced ReSTOR users and had implanted numerous lenses in the preceding 18 months. Patients included were all over 49 years old, with age-related cataract or bilateral presbyopia. They had cataract or presbyopia surgery using phaco-emulsification with IOL implantation in the posterior chamber resulting in post-operative emmetropia. None had refractive surgery before the ReSTOR lens implantation (i.e. LASIK and PRK) or a ReSTOR lens implanted as part of a clinical trial. None had intra-operative difficulties that required them to later wear glasses, post-operative complications necessitating a refractive correction, or concomitant ocular diseases at the time of surgery deteriorating the visual acuity prognosis. Patients meeting the inclusion criteria were selected from an exhaustive list of cases. The target sample included about 150 patients per country. In addition to refining and assessing the psychometric properties of the FGVS, another objective of this study was to find the percentage of ReSTOR IOL patients who do not need glasses 1 year after cataract or presbyopia surgery, and to explore patients' degree of satisfaction with this technology.

The FGVS was administered by phone. Information about patients' use of glasses before and after surgery was collected. Patients' age and gender, clinical characteristics before surgery (general and ocular co-morbidities), vision before surgery, and related information after surgery (posterior capsular opacification, refractive surgery, other ocular diseases) were collected from patient charts.

This study was conducted in compliance with the ethical principles derived from the Declaration of Helsinki, the ADELF (the Association of the French-speaking epidemiologists) and the Good Epidemiology Practices. All patients participating in the study signed a written consent prior to their inclusion. This study received approval of the CNIL (the French data protection permanent committee, authorization N° 907054), the CCTIRS (the French advisory committee dealing with data processing on health research) and the CNOM (the French council of the college of physicians) for France. This study also received approval of the AEMPS (the Spanish agency of drugs and health products) and Comité Ético de Investigación Clínica Regional del Principado de Asturias (the ethical committee of clinical research of the Principality of Asturias) for Spain.

### Statistical analyses

#### Analysis population

The analysis population included all patients for whom the FGVS was completed and considered assessable (i.e. at least 50% of the items were completed). Unless otherwise specified, all statistical analyses were performed on these patients overall as well as by country with a comparison of results between countries.

#### Item reduction and creation of the scoring method for the FGVS

The quality of completion of the FGVS was first analyzed for all FGVS questionnaires received. Item reduction and creation of the scoring method were then conducted using data from patients who completed all FGVS items. Three statistical methods were used to define the structure of the FGVS. First, a Principal Component Analysis (PCA) with varimax rotation was performed to identify the structure of the questionnaire; the retained factors were those with an eigenvalue greater than 1. The structure of the questionnaire was compared between countries, and items not loading on the same scale in both countries were considered for deletion. Second, a multitrait analysis was performed to explore and confirm the structure identified by the PCA. Item convergent and discriminant validity [32] were analyzed (item convergent validity criterion: the correlation between each item and its own hypothesized scale should be higher than 0.40; item discriminant validity criterion: each item should correlate more highly with its own hypothesized scale than with the other scales). Items not loading well on their hypothesized scale were considered for deletion. Lastly, Cronbach's alpha [33] for each scale was analyzed, with a recommended value of at least 0.70 [34]. In addition to using these statistical methods, the final structure of the FGVS was defined based on the content of the items.

#### Psychometric properties of the FGVS

A multitrait analysis was conducted on the analysis population to validate the structure of the FGVS created using the previous analyses. The internal consistency of the FGVS was assessed using Cronbach's alpha for each scale. Percentages at floor and at ceiling were checked to ensure there were no issues related to a high percentage of patients having the lowest or the highest possible score. Known-group validity of the FGVS (the extent to which the FGVS is able to detect variability among patients who are known to differ on various parameters) was analyzed by comparing all scores between subgroups of patients based on wearing glasses before and after surgery, other ocular diseases since surgery, age, gender and country.

#### Statistical tests, level of significance and software

A chi-square test was used when comparing a categorical variable between subgroups of patients. A Mann-Whitney-Wilcoxon test was used when comparing a continuous variable between 2 subgroups of patients. A Kruskal-Wallis test was employed when comparing a continuous variable between more than 2 subgroups of patients. The threshold for statistical significance was fixed at 5%. All analyses were performed using SAS software for Windows (Version 9; SAS Institute, Inc., Cary, NC, USA).

## Results

### Patients' characteristics

Among the 346 patients approached to participate in the study, the 304 patients finally included in the study were kept in the analysis population as they all completed at least 50% of FGVS items. The mean age was 66, with a majority of females (66%). Before surgery, two-thirds of patients had hyperopia (67%), about half had astigmatism (49%) and about a quarter had myopia (28%). About 16% of the patients had ReSTOR implanted for presbyopia. Only 7% of the patients did not have to wear glasses before surgery, whereas 87% were independent from glasses after surgery. A large majority of patients did not develop new eye disease and did not present posterior capsular opacification 1 year after surgery (93% and 85% respectively).

As shown in Table 1, the 152 French and 152 Spanish patients were comparable regarding age, gender, wearing glasses before and after surgery, and development of other ocular disease after surgery. However, statistically significant differences were observed between France and Spain in the percentages of patients with myopia, hyperopia and astigmatism before surgery; there was a higher percentage of patients with myopia and astigmatism in Spain, and a higher percentage of patients with hyperopia in France.

**Table 1:** Patients' characteristics per country (n = 152 for France, n = 152 for Spain).

		France (n = 152)	Spain (n = 152)	p-value of the difference*
***Patients' socio-demographic characteristics***				

Age (years)	Mean (STD)	65 (7.5)	67 (8.9)	0.166
	Median (min - max)	66 (51 - 80)	66 (51 - 83)	

Gender	Males (%)	36	32	0.468

***Patients' characteristics before surgery***				

	Myopia (%)	22	34	***0.010***
Patients' vision**	Hyperopia (%)	74	59	***0.012***
	Astigmatism (%)	35	63	***< 0.001***

	Yes, multifocals (%)	59	60	
Wearing glasses	Yes, single vision (%)	36	30	0.237
	Yes, unknown type (%)	2	1	
	No (%)	4	9	

Best corrected visual acuity	Mean *(STD)*	0.8 (0.2)	0.7 (0.2)	***< 0.001***
	Median *(min - max)*	0.9 (0.3 - 1.2)	0.8 (0.1 - 1.0)	

***Patients' characteristics after surgery***				

	Yes, always (%)	3	7	
Wearing glasses	Yes, sometimes (%)	9	7	0.138
	No/Never (%)	88	86	

Other ocular disease (%)		7	7	0.821

* Chi-square test for categorical variables, Mann-Whitney-Wilcoxon test for continuous variables

In bold and italic, statistically significant p-values

** Myopia, hyperopia and astigmatism may occur simultaneously for some patients, explaining a total percentage by country higher than 100% for patients' vision

### Quality of completion

The quality of completion of the FGVS was very good, with only 9 missing data (3%) observed both for item 17 ("self-image") and item 18 ("other people's image of you"). These missing data were for French patients; no missing data were observed for Spanish patients.

### Distribution of FGVS items

For each of the 21 FGVS items, the majority of patients answered positively (more comfort, less worry, less bother, etc.), with the percentage of positive answers ranging from 51% for item 18 "other people's image of you" to 93% for item 2 "change in life since operation" (Table 2). Spanish patients tended to answer more positively than French patients, whatever the item (percentage of positive answers ranging from 57% to 95% with a mean of 77% for Spanish patients; percentage of positive answers ranging from 39% to 93% with a mean of 69% for French patients). Spanish patients also used the central answer choice more often than French patients (mean percentage of central answer: 18% for Spanish patients; 10% for French patients), whereas French patients used the intermediate answer choices (answers 2 or 4 over a 1-5 range) more often (mean percentage of intermediate answers: 36% for French patients; 11% for Spanish patients).

**Table 2:** Patients' answers to the FGVS items (N = 304).

Content of FGVS^© ^items	Positive answers (%)	Central answer (%)	Negative answers (%)
1. Evaluation of eyesight since operation	88.16	5.59	6.25
2. Change in life since operation	93.09	2.96	3.95
3. Bother of wearing glasses	78.95	6.25	14.80
*Stop wearing glasses because...*			
4. Lenses steaming up	61.18	22.37	16.45
5. Sliding down nose	61.84	21.38	16.78
6. Cleaning lenses	70.07	17.11	12.83
7. Frames restrictive	51.97	22.37	25.66
8. No worry about breaking glasses	59.87	17.76	22.37
9. No worry about scratching glasses	61.18	18.42	20.39
10. No worry about losing glasses	64.14	14.80	21.05
11. Pressing on nose	60.86	19.41	19.74
*Feelings since operation...*			
12. Comfort	89.14	5.26	5.59
13. Freedom	90.79	4.28	4.93
14. Well-being	87.83	6.58	5.59
15. Youth	60.20	21.05	18.75
16. Having new eyes	74.34	11.18	14.47
17. Self-image	70.39	19.74	6.91
18. Other people's image of you	50.66	41.78	4.61
19. Eyesight problems in the past	77.96	11.18	10.86
20. Willingness to undergo surgery again	89.80	2.96	7.24
21. Willingness to recommend to others	88.49	3.95	7.57

### Item reduction and creation of the scoring method for the FGVS

The item reduction was conducted using data from the 295 patients who completed all FGVS items (143 in France and 152 in Spain). As shown in Table 3, the PCA conducted on the 21 FGVS items resulted in four factors with an eigenvalue greater than 1, explaining 66% of the total variance. Item 15 ("feel younger since operation") was deleted from the questionnaire as it loaded on several scales with the global PCA, and did not load on the same scale with PCAs conducted on French and Spanish patients separately. The final structure of the questionnaire, presented in Table 4, uses 20 of the 21 original FGVS items. Items 3 ("bother of wearing glasses"), 17 ("self-image") and 18 ("other people's image of you"), corresponding to the fourth factor of the PCA, were kept but not aggregated in a dimension. Item 3 loaded on several scales. The content of items 17 and 18 was considered off-topic as they did not refer to the surgery or advantages of freedom from glasses, and missing data were observed for these 2 items in French patients. However, as these 3 items might be good predictors of the patients' motivation for surgery, these items were retained in the questionnaire. The other 17 items were grouped into 2 dimensions ('global evaluation', 9 items; 'advantages', 8 items). This structure, based on the results of the PCA, was confirmed by the results of the multitrait analysis, with all items meeting both the convergent and discriminant validity criteria, and the Cronbach's alpha values of each dimension exceeding 0.70. The 2 dimensions were then divided into 5 sub-dimensions based on the content of the items: 'global evaluation' was divided into 'evaluation of the result' (2 items), 'feelings' (4 items) and 'global judgment' (3 items); 'advantages' was divided into 'psychological advantages' (3 items) and 'practical advantages' (5 items). The results of the multitrait analysis for these 5 sub-dimensions were good, with all items meeting the convergent validity criterion and only a few items not meeting the discriminant validity criterion. The Cronbach's alpha was above 0.70 for the 5 sub-dimensions. Scale-scale correlations between the 5 sub-dimensions ranged between 0.27 and 0.66, indicating a link but no redundancy between the sub-dimensions, reinforcing the decision to keep them in the final structure of the FGVS.

**Table 3:** Results of the PCA conducted on FGVS items (N = 295).

	Factor 1	Factor 2	Factor 3	Factor 4
Eigenvalue for each factor	8.51	2.98	1.32	1.11
Proportion of variance explained by each factor	0.41	0.14	0.06	0.05

Factor loadings for each item:				

2. Change in life since operation	**0.84**	0.21	0.03	0.07
12. *Feelings since operation... *Comfort	**0.84**	0.14	0.22	0.20
20. Willingness to undergo surgery again	**0.83**	0.11	0.01	0.01
14. *Feelings since operation... *Well-being	**0.82**	0.11	0.18	0.30
13. *Feelings since operation... *Freedom	**0.82**	0.08	0.17	0.26
19. Eyesight problems in the past	**0.76**	0.11	0.15	0.10
21. Willingness to recommend to others	**0.76**	0.07	0.07	0.06
1. Evaluation of eyesight since operation	**0.73**	0.23	-0.03	-0.07
16. *Feelings since operation... *Having new eyes	**0.68**	0.13	0.20	0.25
15. *Feelings since operation... *Youth	**0.55**	0.12	0.13	0.41

10. *Stop wearing glasses because... *No worry about losing glasses	0.14	**0.86**	0.10	0.15
8. *Stop wearing glasses because... *No worry about breaking glasses	0.21	**0.83**	0.22	0.10
9. *Stop wearing glasses because... *No worry about scratching glasses	0.17	**0.80**	0.33	0.10
11. *Stop wearing glasses because... *Pressing on nose	0.15	**0.57**	0.27	0.19
7. *Stop wearing glasses because... *Frames restrictive	0.20	**0.51**	0.44	0.11

5. *Stop wearing glasses because... *Sliding down nose	0.11	0.20	**0.79**	0.16
6. *Stop wearing glasses because... *Cleaning lenses	0.17	0.29	**0.78**	0.13
4. *Stop wearing glasses because... *Lenses steaming up	0.09	0.33	**0.74**	-0.05

17. Self-image	0.20	0.13	0.00	**0.83**
18. Other people's image of you	0.14	0.21	0.09	**0.69**
3. Bother of wearing glasses	-0.14	-0.09	-0.41	**-0.50**

**Table 4:** Final structure of the FGVS.

Item		Sub-dimension	Dimension
		
Number	Label		
1	Evaluation of eyesight since operation	Evaluation of the result	
2	Change in life since operation		
	
	*Feelings since operation*		
12	... Comfort		
13	... Freedom	Feelings	
14	... Well-being		Global evaluation
16	... Having new eyes		
	
19	Eyesight problems in the past		
20	Willingness to undergo surgery again	Global judgment	
21	Willingness to recommend to others		

	*Stop wearing glasses because*		
4	... Lenses steaming up		
5	... Sliding down nose	Practical	
6	... Cleaning lenses	advantages	
7	... Frames restrictive		Advantages
11	... Pressing on nose		
	
8	... No worry about breaking glasses		
9	... No worry about scratching glasses	Psychological	
10	... No worry about losing glasses	advantages	

3	Bother of wearing glasses		
17	Self-image	*Not aggregated in a dimension*	
18	Other people's image of you		

In terms of calculation of scores, all item scales were reversed so that a higher item score reflects a more positive answer. A score was then calculated for each dimension and sub-dimension as the mean of reversed items in the dimension/sub-dimension. All dimension and sub-dimension scores ranged from 1 to 5, with a higher score indicating a more positive evaluation and greater advantages of freedom from glasses. To handle missing data, sub-dimension scores were calculated if at least 1 item of the sub-dimension was completed, and dimension scores were calculated only if all corresponding sub-dimensions were scored.

### Psychometric properties of the FGVS

As presented in Table 5, the multitrait analyses conducted on the overall analysis population to validate the 2 dimensions and 5 sub-dimensions of the FGVS showed that all items met the convergent validity criterion. Only a few items did not meet the discriminant validity criterion (1 item in the 'evaluation of the result' sub-dimension, 1 item in the 'global judgment' sub-dimension and 1 item in the 'practical advantages' sub-dimension). The Cronbach's alpha was higher than the recommended 0.70 threshold for the 2 dimensions (0.93 for 'global evaluation' and 0.89 for 'advantages'), as well as for all sub-dimensions (ranging from 0.78 for 'evaluation of the result' to 0.91 for 'feelings'), indicating good internal consistency reliability. A ceiling effect was observed for the 2 dimension scores (32.2% for 'global evaluation' and 18.4% for 'advantages'), as well as for all sub-dimension scores (ranging from 20.1% for 'psychological advantages' to 56.6% for 'evaluation of the result'). No floor effect was observed, either for the 2 dimension scores or for the 5 sub-dimension scores. Similar results were found in France and Spain.

**Table 5:** Psychometric validation of the final structure of the FGVS (N = 304).

	Number of items	% at floor	% at ceiling	Range of Spearman item-scale correlations	% of items meeting the convergent validity criterion	% of items meeting the discriminant validity criterion*	Cronbach's alpha
**Evaluation of the result**	2	0.3	56.6	0.57 - 0.57	100%	50%	0.78
**Feelings**	4	2.0	47.7	0.66 - 0.77	100%	100%	0.91
**Global judgment**	3	2.0	55.3	0.54 - 0.60	100%	67%	0.84

***Global evaluation***	*9*	*0.3*	*32.2*	*0.52 - 0.75*	*100%*	*100%*	*0.93*

**Practical advantages**	5	1.0	20.1	0.55 - 0.65	100%	80%	0.82
**Psychological advantages**	3	3.3	40.8	0.79 - 0.85	100%	100%	0.89

***Advantages***	*8*	*0.3*	*18.4*	*0.58 - 0.81*	*100%*	*100%*	*0.89*

* For each item, the correlation coefficient with its own scale is compared to correlation coefficients with the other scales. The percentage of items for which the correlation with its own scale is greater than the correlation with all the other scales is reported.

Patients not wearing glasses after surgery reported higher 'global evaluation' and 'advantages' dimension scores (mean score of 4.6 and 3.9 respectively), indicating a better global evaluation and greater advantages than patients wearing glasses (mean scores lower than 4.0 and 3.5, respectively). As shown in Table 6, these differences were statistically significant, and the differences in the 5 sub-dimension scores were also statistically significant between those patients, with higher scores for patients not wearing glasses after surgery. Patients with no ocular co-morbidity after surgery reported a higher 'global evaluation' score (mean 4.5), indicating a better global evaluation, than those with an ocular co-morbidity after surgery (n = 21, mean score of 3.8). This difference was statistically significant. Regarding the 'global evaluation' dimension, only the difference in the 'evaluation of the result' sub-dimension score was statistically significant between those patients, with a higher score for patients who did not develop another ocular disease after surgery. The comparison of the FGVS sub-dimension and dimension scores between groups of patients based on the wearing of glasses before surgery, age and gender had non-significant results. Statistically significant differences in 'global evaluation' and 'advantages' dimension scores and the 5 sub-scores were observed between France and Spain, with higher scores for Spanish patients, indicating a better global evaluation and greater advantages.

**Table 6:** Known-group validity: results for groups presenting a statistically significant difference in FGVS 'global evaluation' and/or 'advantages' score(s) (N = 304).

			Global evaluation scores				Advantages scores		
			
				Sub-dimension scores				Sub-dimension scores	
						
			Dimension score	Evaluation of the result	Feelings	Global judgment	Dimension score	Practical advantages	Psychological advantages
**Wearing glasses after surgery**	Yes, always	Mean (STD)	3.6 (1.4)	3.9 (1.2)	3.5 (1.6)	3.6 (1.4)	3.5 (1.0)	3.6 (1.0)	3.3 (1.3)
	(N = 15)	Median	4.6	4.5	4.8	4.3	3.8	3.6	3.0
	Yes, sometimes	Mean (STD)	4.0 (1.0)	4.2 (0.7)	3.9 (1.1)	3.9 (1.2)	3.3 (1.0)	3.3 (0.9)	3.3 (1.3)
	(N = 24)	Median	4.2	4.0	4.4	4.3	3.0	3.1	3.0
	No/Never	Mean (STD)	4.6 (0.7)	4.6 (0.7)	4.5 (0.8)	4.6 (0.8)	3.9 (1.0)	3.9 (1.0)	3.9 (1.2)
	(N = 265)	Median	4.8	5.0	5.0	5.0	4.0	4.0	4.0
	
	*p-value*		<0.001	<0.001	<0.001	<0.001	0.006	0.007	0.017

Other ocular disease since surgery*	Yes	Mean (STD)	3.8 (1.6)	3.9 (1.3)	3.6 (1.7)	3.8 (1.7)	3.4 (1.1)	3.5 (1.1)	3.3 (1.4)
	(N = 21)	Median	4.7	4.5	4.8	4.7	3.3	3.6	3.0
	No	Mean (STD)	4.5 (0.7)	4.6 (0.7)	4.5 (0.8)	4.5 (0.8)	3.8 (1.0)	3.8 (1.0)	3.8 (1.2)
	(N = 283)	Median	4.8	5.0	4.8	5.0	4.0	4.0	4.0
	
	*p-value*		0.044	0.023	*NS*	*NS*	*NS*	*NS*	*NS*

* All 21 patients reported a different ocular disease since surgery, including for example conjunctivitis, problems with tears, sty or retinal detachment

Presented p-values are p-values of the difference between groups (Mann-Whitney-Wilcoxon or Kruskal-Wallis test); NS, Not Significant

## Discussion

The FGVS, which measures benefits of freedom from glasses perceived by cataract and presbyopic patients after multifocal IOL surgery, was administered to French and Spanish patients who had had a ReSTOR lens implanted in both eyes, with the last lens implanted at least 1 year before inclusion in the study. The large majority of patients included in the study had ReSTOR implanted for cataracts. Subjective refraction parameters before surgery were not collected and the high hyperopia incidence rate is likely to include a majority of mild hyperopia. This high rate of hyperopia might influence the spectacle independence rate. This possible selection bias can only be definitely addressed using a randomized clinical trial. The FGVS was well accepted by all patients as very few missing data were observed. Only a small percentage of French patients did not answer when they were asked about their physical appearance and aesthetic aspects (i.e. preference with or without glasses).

The final version of the FGVS showed good psychometric properties, including good item convergent and discriminant validity and good internal consistency reliability of the dimensions as well as sub-dimensions. The FGVS was also able to discriminate between patients according to the wearing of glasses after surgery and according to the occurrence of another ocular disease after surgery. Patients not wearing glasses after surgery reported a more positive evaluation and greater advantages of freedom from glasses than those who had to wear glasses, and patients who had no other ocular disease after surgery reported a more positive evaluation of surgery than those who had ocular disease. Similar psychometric results were observed for France and Spain. These results demonstrate the validity of the final structure of the FGVS.

Along with good psychometric results, a noteworthy result of this study was the difference observed between French and Spanish patients in their response patterns. Spanish patients tended to answer more positively than French patients to all FGVS items, and were more likely to use the central answer choice than French patients. French patients were more likely to use the intermediate answer choices. Spanish patients tended to report a better global evaluation of surgery and greater advantages of freedom from glasses than French patients. This produced a statistically significant difference between countries in both scores. Three arguments could be put forward to explain these differences: issues with cross-cultural performances of the French and Spanish versions of the FGVS, differences in the clinical status of French and Spanish patients, or cultural and social differences between France and Spain. First, one could argue that the differences observed between French and Spanish patients could be due to issues related to the cross-cultural validity of the FGVS, such as construct or item bias. However, the PCA found that the 2 versions had similar structures, and a quasi-systematic difference in the item responses was observed, indicating that issues with the cross-cultural validity of the FGVS is unlikely to be the major cause of these differences. Another explanation could be the difference in patients' clinical status. However, except in the proportion of myopic, astigmatic and hyperopic patients, French and Spanish patients were comparable, especially regarding the use of glasses before and after surgery. The most plausible explanation of the difference in the response patterns between French and Spanish patients in our study is the response style bias, defined as "a systematic tendency to respond to a range of questionnaire items on some other basis than the specific item content" [35]. Two highly problematic issues in attitude and survey research regarding response style bias are acquiescence and extreme response style [36,37]. In 2004, van Herk et al [38] showed that Spanish respondents tended to display higher acquiescence and to use more extreme responses than did French respondents, and our results are in line with those findings. The influence of some cultural characteristics of a society on the way that questions are answered has been explored and is well-known [39,40]. Thus the differences observed between French and Spanish patients in the present study, reinforced by the complexity of the role played by cultural features in the response style bias, suggest that the country effect should be systematically tested when using the FGVS.

A ceiling effect was observed for the 'global evaluation' score, which could be considered a limitation of our study. However, our study involved only patients who had no complications during surgery that required them to wear glasses and no post-surgery infectious complications. Therefore, all patients included were likely to be globally satisfied with surgery, and results may be different in a study based on a prospective study design. Another limitation of the present study is that some important psychometric properties, including concurrent validity, as well as reproducibility (test-retest reliability) and responsiveness, could not be assessed because the study design used only one PRO instrument and one evaluation. The psychometric validation of the FGVS should therefore be completed by using the questionnaire in another study in conjunction with other PRO instruments and with several different time point assessments. In addition, the questionnaire was administered by phone in our study; in future research the acceptability of the FGVS should also be analyzed when administered using another mode of administration. Lastly, additional work should assess the usefulness of the 3 items that were kept in the questionnaire but that did not load on either domain.

One could argue that our patient cohort was a mixed group of individuals suffering from different eye conditions which could affect patients' outcomes. Although patients with presbyopia might have different expectations than patients with cataract, the number of patients with presbyopia included in our survey was not big enough to allow any inferences. Moreover, the question of the relation between factors such as the age, gender or medical history, and expectations is still not resolved. Additional research with specific experimental designs should be conducted to confirm these findings.

In our study design, all FGVS items were completed after surgery. However, the structure of the final version of the FGVS, measuring on the one hand the advantages of no longer wearing glasses and evaluating on the other hand patients' satisfaction with the IOL surgery, suggests another potential future use of the questionnaire. The first part of the questionnaire on the advantages of freedom from glasses could be answered by patients before surgery in order to assess their expectations, while the second part on patients' satisfaction could be answered by patients after surgery in order to assess patients' satisfaction. Thus, the first part could be used for different purposes: first to describe the baseline expectations of patients, then as a covariate in the analysis of the benefit of surgery as measured by the 'global evaluation' score, and finally, alongside other clinical criteria, to document the patient's eligibility for surgery.

The final structure of the FGVS provides an overall view with its two 'global evaluation' and 'advantages' dimensions, as well as a more subtle view and particular perspective with its five sub-dimensions. Therefore, we recommend using a hierarchical process when analyzing FGVS data, by first evaluating the two dimension scores, and then, depending on the results obtained for dimension scores, evaluating the five sub-dimension scores.

## Conclusions

Even though further validation analyses would be useful to complete the present study, the final version of the FGVS proved to be a reliable and valid PRO instrument that enables the practical and psychological advantages of freedom from glasses and patient satisfaction after multifocal IOL surgery to be assessed.

## Competing interests

The present work was funded by Alcon, France SA, Rueil-Malmaison. Benoit Arnould, Juliette Meunier and Muriel Viala-Danten are paid consultants to Alcon. Gilles Berdeaux is an employee of Alconlabs.

## Authors' contributions

All authors provided intellectual contributions to this manuscript. GB conceived the study and participated in the interpretation of data. JM designed and programmed statistical analyses, and participated in the interpretation of data. BA provided input in the interpretation of data and reviewed this manuscript. MVD participated in the design of statistical analyses and interpretation of data. All authors read and approved the final manuscript.

## Pre-publication history

The pre-publication history for this paper can be accessed here:

http://www.biomedcentral.com/1471-2415/10/15/prepub
